# Prospective Associations Between Salivary Biomarkers of Inflammation and Body Mass Index in Adolescents

**DOI:** 10.1002/osp4.70081

**Published:** 2025-06-21

**Authors:** Keri M. Kemp, Catheryn A. Orihuela, Douglas A. Granger, Retta R. Evans, Sylvie Mrug

**Affiliations:** ^1^ Division of Gerontology, Geriatrics and Palliative Care Department of Medicine Heersink School of Medicine University of Alabama at Birmingham Birmingham Alabama USA; ^2^ Department of Family and Community Medicine Heersink School of Medicine University of Alabama at Birmingham Birmingham Alabama USA; ^3^ Institute for Interdisciplinary Salivary Bioscience Research University of California Irvine California USA; ^4^ Department of Pediatrics Johns Hopkins University School of Medicine Baltimore Maryland USA; ^5^ Department of Human Studies School of Education and Human Sciences The University of Alabama at Birmingham Birmingham Alabama USA; ^6^ Department of Psychology University of Alabama at Birmingham Birmingham Alabama USA

**Keywords:** adolescence, inflammation, obesity, salivary biomarkers

## Abstract

**Background:**

Childhood and adolescent obesity, which affects nearly 1 in 5 youth in the US, presents a pressing public health concern. Obesity is linked to chronic low‐grade inflammation, which exacerbates comorbidities. Noninvasive tools are needed to monitor obesity‐related inflammation and assess weight‐management interventions in children and adolescents.

**Objective:**

This study investigated the associations between Body Mass Index z‐score (BMIz) and salivary biomarkers: C‐reactive protein (CRP), cytokines interleukin (IL)‐1β, IL‐6, IL‐8, and tumor necrosis factor (TNF)‐α.

**Methods:**

A sample of 280 adolescents (Mage = 12.1 years, SD = 0.44) was followed for 2 years (3 time points) from 2019 to 2021. An autoregressive cross‐lagged path model was used to examine the prospective relationships between BMIz and salivary biomarkers.

**Results:**

Findings indicated a bidirectional relationship between BMIz and salivary CRP levels, suggesting a feed‐forward cycle in which excessive weight gain and inflammation mutually amplify each other. Salivary cytokines were not associated with BMIz.

**Conclusions:**

This study underscores the utility of salivary CRP as a noninvasive biomarker for obesity‐related inflammation. Monitoring salivary CRP levels could aid in targeting interventions to prevent obesity‐related complications early in life.

## 1 Introduction

Obesity affects nearly 14.7 million children and adolescents in the United States [1], presenting a tremendous public health challenge. Almost 80% of adolescents with obesity will continue to experience obesity as adults [2] and face an increased risk of morbidity and mortality [3]. Low‐grade inflammation triggered by excessive weight gain is a critical factor in developing obesity‐related conditions, including cardiovascular disease, type 2 diabetes, insulin resistance, polycystic ovary syndrome, and osteoarthritis [4]. Therefore, reducing inflammation to prevent complications is often a key goal when treating obesity [5]. Noninvasive measures of inflammation could aid in monitoring individual responses to interventions and guide personalized treatment approaches aiming to reduce inflammation and improve metabolic health [6]. This study examined the utility of salivary biomarkers as a non‐invasive means to assess systemic inflammation related to obesity during early adolescence.

The relationship between adiposity and inflammation is complex and bidirectional. Activation of the innate immune system and macrophage infiltration of adipose tissue occurs early during excess weight gain. Once recruited into the adipose tissue, immune cell activation and polarization occur because of direct cell‐to‐cell contact with adipocytes or through secreted molecules. This cascades into the secretion of proinflammatory mediators, including interleukin (IL)‐6, IL‐8, IL‐1β, and tumor necrosis factor‐alpha (TNF‐α), into the circulation [4, 7]. The accumulation of dysfunctional adipose tissue results in adipocyte apoptosis or death, metabolic stress, and hypoxia and exacerbates the secretion of proinflammatory cytokines in circulation [8, 9].

Conversely, inflammation can also promote the development and persistence of obesity. Elevated levels of proinflammatory cytokines stimulate the liver to produce C‐reactive protein (CRP), perpetuating low‐grade systemic inflammation [10]. Furthermore, insulin resistance can develop when proinflammatory cytokines interfere with insulin signaling pathways and adipogenesis [11].

Despite strong evidence linking obesity and inflammation, gaps remain in understanding this relationship in early adolescence. Studying the interplay between adiposity and inflammation during this period can provide valuable insights into the early onset of the obesity‐inflammation cycle and inform preventive strategies. Most studies have focused on adults or utilized cross‐sectional designs, limiting the ability to infer causality or the directionality of these associations in children and young adolescents. Early adolescence is a critical developmental period characterized by rapid physical, psychological, and hormonal changes, which can significantly influence body composition and metabolic regulation [12, 13].

Sex differences have been reported in the expression patterns of inflammatory cytokines in children with obesity, with females presenting a lower inflammatory status compared with males [14]. Animal models of obesity also found a general trend of lower obesity‐related inflammation in females compared with males [15]. These findings highlight the importance of considering sex as a biological variable when studying obesity‐related inflammation.

Most research relies on monitoring obesity‐associated inflammation through blood samples collected in a clinical setting by a trained phlebotomist. Collecting blood samples in children is complicated, with higher failure rates for sticks, and can cause the child fear and anxiety [16]. There is a need for noninvasive tools that can be easily implemented in nonclinical settings to monitor the effectiveness of obesity treatments in reducing systemic inflammation and identifying individuals at a greater risk for obesity‐related comorbidities [5].

Saliva biomarkers offer a promising avenue for assessing the efficacy of obesity treatment, particularly in vulnerable populations such as children, because of their noninvasive collection, simple handling, and storage [17]. For example, CRP is stable for 8 hours at room temperature in saliva collected by passive drool [18]. This approach enables easy sampling even in non‐clinical settings such as school classrooms, facilitating broader research and intervention efforts [19].

However, the use of salivary cytokines to monitor inflammatory status in children and adolescents with obesity is in its early stages. Cross‐sectional studies have reported mixed results, either finding positive associations between salivary markers of inflammation (CRP, IL‐1β, IL‐6, IL‐8, and TNF‐α) and measures of obesity or no association [20, 21, 22, 23, 24]. Moreover, there is a lack of longitudinal studies examining the bidirectional relationships between weight gain and inflammation in children and adolescents [20]. It is still unclear if body mass index (BMI) predicts increased inflammation over time as measured by salivary markers. Additional investigations are needed to evaluate the utility of salivary biomarkers of inflammation as a tool for predicting future weight gain in children and adolescents before it can be used as a tool to assess the efficacy of interventions [25].

Previous studies have often been cross‐sectional, limited to racially and ethnically homogeneous samples, and conducted in clinical settings [18, 21, 24]. This study expands on these findings by examining the bidirectional relationships between body mass index (BMI) and salivary markers (CRP, IL‐1β, IL‐6, IL‐8, and TNF‐α) over one to 2 years in a diverse group of adolescents from the community. Additionally, this study includes sex as a biological factor and applies findings to non‐clinical settings where blood collection technology and expertise are often lacking.

## 2 Methods

### 2.1 Sample

**FIGURE 1:**
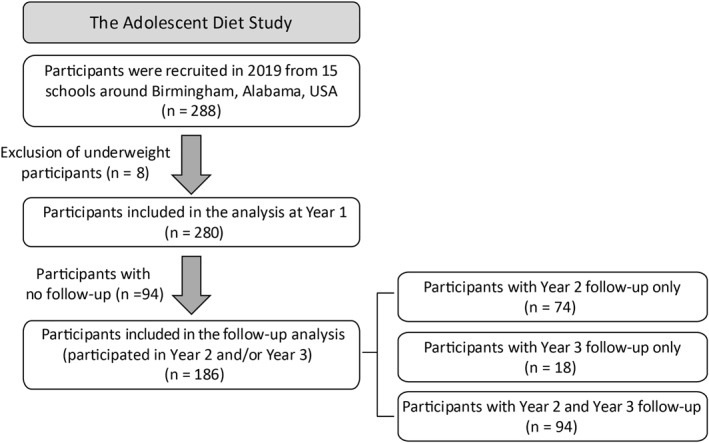
Study sample.

This study focused on early adolescents who participated in The Adolescent Diet Study, a school‐based, long‐term examination of adolescent health (Figure 1). Adolescents from 6th or 7th grade in 15 different schools in the greater Birmingham, AL area were recruited for the study. A total of 288 students (Mage = 12.01, SD = 0.44; 54% female; 48% Black, 37% White, 10% Hispanic, and 5% Other race and ethnicity) were recruited in Year 1. Participants classified as underweight (BMIz < 5th percentile) in Year 1 were excluded, leaving an analytic sample of 280 participants. These adolescents were reassessed in Year 2 (12 months later in 2020) and Year 3 (14 months after Year 2 in 2021). The COVID‐19 pandemic, which caused school closures, adversely affected retention rates, with 60% (*n* = 167) of the sample retained in Year 2% and 40% (*n* = 112) in Year 3. Only 51% (*n* = 94) of participants with follow‐up data participated in both Year 2 and Year 3 assessments. Therefore, to maximize sample size at follow‐up, Year 2 and Year 3 data were aggregated into a single variable (Year 2/3), averaging across Years 2 and 3 if both were available. This resulted in 186 participants with follow‐up (Year 2/3) data (Figure 1).

### 2.2 Procedure

Students were recruited from one or two classrooms per school between January and December 2019. All English‐speaking children capable of completing the study activities were invited, resulting in a participation rate of 45%. During Year 1 (2019), data collection occurred at the school on regular weekdays, Monday through Friday. School closures and visitor restrictions during the COVID‐19 pandemic in Years 2 and 3 (2020 and 2021) necessitated a shift in study procedures to biosample collection at home and interviews held at a university laboratory. Participants received monetary compensation for each year of their involvement in the study.

### 2.3 Measures

#### 2.3.1 Body Mass Index (BMI)

Height and weight were measured by trained research staff using a stadiometer and a scale. Before measurements, adolescents were asked to remove shoes and bulky clothing, such as jackets or sweaters. Two readings were recorded for height and weight to the nearest 0.01 kg or 0.10 cm. If the difference between the readings exceeded 0.20 kg or 0.50 cm, a third reading was taken, and the two closest values were averaged. Standardized BMI (BMIz) was calculated using the 2022 Centers for Disease Control and Prevention (CDC) extended BMI‐for‐age growth charts for children and adolescents with very high BMIs [26].

#### 2.3.2 Salivary Biomarkers

To minimize the influence of circadian rhythms on salivary biomarkers [17], saliva samples were collected at approximately 8:00 a.m. on four consecutive days (Monday–Thursday) in Year 1 and Year 2 of the study. In Year 3, samples were collected on a single day. To eliminate food particles, students were instructed to rinse their mouths with water and wait 10 min before providing 1 mL of saliva through the passive drool technique [27]. Samples were kept on ice or in a home freezer and then transferred on ice to a university lab where they were immediately stored in a −80°C freezer until analyses.

Samples were analyzed in duplicate for CRP with the Salimetrics Salivary CRP Enzyme Immunoassay Kit (item: 1‐2102) and in singlets for IL‐1β, IL‐6, IL‐8, and TNF‐α with the Salimetrics Salivary Cytokine Panel (item: 5209.25). Samples above and below the standard curve were winsorized by replacing the datapoint with the highest value in the standard curve or the value for the lower detection limit, rounded to 0.01 pg/mL [28, 29]. The CRP technical duplicates were averaged to obtain the sample mean. Biomarker data were then log‐transformed to correct for positive skew. The distributions of transformed data were examined with histograms and the degree of kurtosis and skewness was calculated with the R packages rcompanion (version 2.4.36) [30] and moments (version 0.14.1) [31]. Based on prior approaches to reduce the influence of outliers [28, 32, 33], observations > ± 3.5 standard deviations (SD) from the mean were winsorized to the nearest 0.01 pg/mL.

For each participant, the four daily sample measurements were averaged within an assessment year to derive a single value for Year 1 and a single value for Year 2. The reliability of salivary biomarkers was assessed by calculating the within‐subject (individual) coefficient of variation (CV_I_) as the standard deviation (SD) divided by the mean of biomarker measurements across the four sampling days within a year. Then, the mean CV_I_ and 95% confidence interval were calculated for each biomarker for each year. The Intraclass Correlation Coefficient (ICC) was calculated using a linear mixed‐effects model with a random intercept for participant and a fixed intercept estimating the average concentration of the biomarker across all participants, fitting the biomarker concentration as the dependent variable. To estimate the ICC 95% confidence interval, bootstrapping (1000 resamples) was applied for each biomarker. ICC values were interpreted as follows: < 0.5, poor reliability; ≥ 0.5–0.75, moderate reliability; ≥ 0.75–0.9, good reliability; and ≥0.9 excellent reliability [34].

#### 2.3.3 Covariates

In Year 1, parents reported annual household income, rated on a 13‐point scale (1 = less than $5,000, 13 = more than $90,000), and their highest educational attainment on a 7‐point scale (1 = no high school diploma, 7 = graduate or professional degree). SES was created as a composite score calculated from the average standardized income and parental education (*r* = 0.51, *p* < 0.001). Parents provided information on their adolescent's sex (coded 0 for male, 1 for female) and race and ethnicity. The study sample consisted primarily of non‐Hispanic White and Black participants (84%), with a lower representation of individuals identifying as Hispanic or another racial and ethnic group. Thus, the sample sizes of non‐Black and non‐White racial/ethnic groups were too small to analyze as separate groups. In the US population, obesity prevalence is similarly high among Black (24.8%) and Hispanic children (26.2%) compared with non‐Hispanic White Children (16.6%) [35]. Therefore, race and ethnicity were coded as a binary variable (0 for non‐Hispanic White, 1 for Black, Hispanic, or other) to improve statistical power. Given that race and ethnicity was used as a covariate and not a variable of main interest, this approach was deemed acceptable. Finally, a time variable was calculated and included as a covariate to account for individual differences in the time elapsed between Year 1 and the follow‐up. This variable was calculated in the same manner as the follow‐up biomarker data by aggregating across years 2 and 3 and calculating the mean time elapsed if both years were available.

### 2.4 Statistical Analyses

#### 2.4.1 Preliminary Analyses

Data cleaning and preliminary analyses were conducted in *RStudio version 3.4.0* [36]. Attrition analyses compared participants with any follow‐up data (Year 2, Year 3, or both years) to those missing follow‐up data across all baseline variables with Mann‐Whitney U and Chi‐square tests. Bivariate correlations among key study variables were examined using Spearman's ⍴. Due to a high degree of missingness, salivary biomarker levels were aggregated into a single follow‐up variable (Year 2/3). Prior to aggregation, Wilcoxon rank‐sum tests were performed to evaluate differences in salivary biomarker levels between Year 2 and Year 3 assessments, while Levene's tests were used to confirm variance homogeneity across the two assessment years.

#### 2.4.2 Main Analyses

**FIGURE 2:**
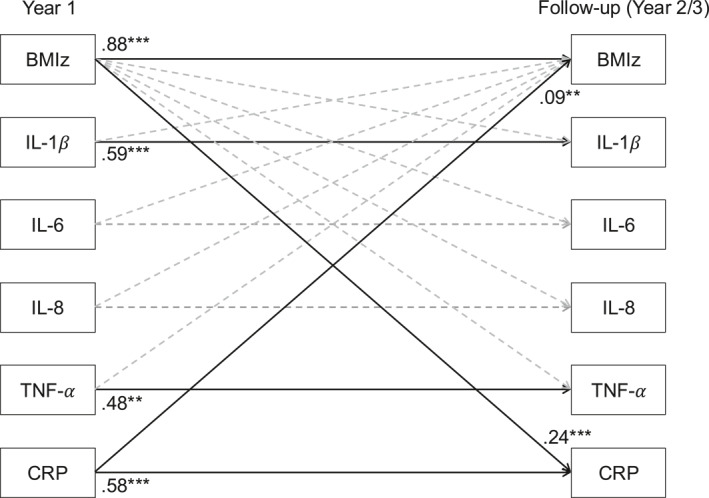
Autoregressive cross‐lagged model of prospective bidirectional relationships between standardized body mass index (BMIz) and salivary biomarkers of inflammation: C‐reactive protein (CRP), interleukin (IL)‐1β, IL‐6, IL‐8, tumor necrosis factor (TNF‐α). All paths were adjusted for age, sex, SES, and time elapsed between Year 1 and follow‐up. All variables within each time point were allowed to covary. Solid lines indicate significant paths (*p* < 0.05), whereas dashed lines indicate nonsignificant paths. Standardized coefficients are shown for significant paths. **p ≤ 0.01; ***p ≤ 0.001.

Prospective bidirectional relationships between BMIz and salivary biomarkers were analyzed with an autoregressive cross‐lagged path model in Mplus version 8.1 [37]. Missing data (15.3% of data points) were addressed using Full Information Maximum Likelihood (FIML). FIML was utilized because it outperforms other approaches to missing data [38]. FIML utilizes all available data, produces unbiased estimates and standard errors when data are missing at random, and maintains the total sample size (*N* = 280) [38, 39]. The model included autoregressive paths between the same variables at Year 1 and Year 2/3 follow‐up and cross‐lagged paths prospectively linking BMIz at Year 1 with salivary biomarkers at follow‐up and each salivary biomarker from Year 1 with BMIz at follow‐up. In FIML, the inclusion of too many free parameters can make it difficult to estimate a unique solution. Therefore, to enhance model identification, reduce complexity, and improve stability and interpretability, cross‐lagged links among salivary biomarkers and between race/ethnicity and biomarkers were omitted (see Figure 2). All variables measured at the same time point were allowed to covary. Sex, age, SES, and time passed between Year 1 and follow‐up were included as covariates. Multiple‐group analysis was conducted to determine if the relationships between BMIz and salivary inflammation markers differed between females and males. A constrained model that fixed the prospective paths between BMIz and inflammatory markers to be the same for both sexes was compared with an unconstrained model that allowed these paths to vary between sexes. A significant likelihood ratio test between these two models indicates that sex differences exist in the association between BMIz and inflammation.

### 2.5 Ethics

This study was conducted following the guidelines established in the Declaration of Helsinki. All procedures involving human subjects or patients were approved by the University of Alabama at Birmingham Institutional Review Board (IRB 300002344). Written informed consent was obtained from all participants.

## 3 Results

### 3.1 Preliminary Analyses

Table 1 presents sociodemographic data for the sample. Most of the participants were either Black (48%) or non‐Hispanic White (36%). The sample was socioeconomically diverse, with a median household income ranging from $30,000 to $50,000 and a median parental education level of “some college but no degree.” Attrition analyses revealed that participants lacking follow‐up data had lower BMIz in Year 1 (*M* = 0.8, SD = 1.15) compared to those with follow‐up data (*M* = 1.2, SD = 1.12; *U* [*N*_no follow‐up_ = 94, *N*_follow‐up_ = 185] = 7183, *p* = 0.017). Participants without follow‐up data were also less likely to identify as a minority race or ethnicity (53%) compared to those with follow‐up data (69%; *χ* ^*2*^ [1, *N* = 276] = 6.77, *p* = 0.009). There were no differences in age, sex, and SES between participants with and without follow‐up data (*p* > 0.05).

**TABLE 1:** Sample demographics.

	Year 1	Year 2	Year 3
Characteristic	*N* = 280^a^	*N* = 168^a^	*N* = 112^a^
Age (years)	12.1 (0.44)	13.2 (0.45)	14.2 (0.46)
Female	152 (54%)	87 (52%)	58 (52%)
Race and ethnicity			
White	99 (36%)	52 (31%)	37 (33%)
Black	133 (48%)	87 (52%)	57 (51%)
Hispanic	29 (11%)	21 (13%)	12 (11%)
Other race and ethnicity	15 (5.4%)	8 (4.8%)	6 (5.4%)
*Missing*	4	0	0
BMIz	1.1 (1.14)	1.3 (1.06)	1.0 (1.11)
*Missing*	1	9	6
Weight category			
Healthy weight	133 (48%)	67 (42%)	57 (54%)
Overweight	54 (19%)	34 (21%)	16 (15%)
Obesity	52 (19%)	28 (18%)	17 (16%)
Severe obesity	19 (6.8%)	12 (7.5%)	7 (6.6%)
Very severe obesity	21 (7.5%)	18 (11%)	9 (8.5%)
*Missing*	1	9	6
Annual parental income			
< $10,000	29 (12%)	17 (11%)	12 (12%)
$10.001–50,000	99 (42%)	61 (40%)	41 (39%)
$50.001–90,000	47 (20%)	35 (23%)	22 (21%)
> $90,000	61 (26%)	38 (25%)	29 (28%)
*Missing*	44	17	8
Parental education			
< 12th grade	25 (9.8%)	14 (8.8%)	7 (6.5%)
High school diploma or GED	47 (19%)	26 (16%)	18 (17%)
Some college or associate degree	95 (37%)	63 (40%)	43 (40%)
Bachelor's degree	50 (20%)	35 (22%)	22 (20%)
Graduate or professional degree	37 (15%)	21 (13%)	18 (17%)
*Missing*	26	9	4

Abbreviations: BMIz, standardized body mass index; GED, general education degree.

^a^ Mean (SD); *n* (%).

Salivary biomarker samples flagged as ‘non‐detects' were winsorized by replacing the datapoint with the next highest or lowest value from values within the standard curve range, rounded to 0.01 pg/mL. All IL‐1β, IL‐6, and IL‐8 samples were within the detectable range. For CRP, 0.25% (*n* = 8) of samples were below, and 0.65% (*n* = 21) were above the detectable range. Only one TNF‐α sample fell below the detectable range and no TNF‐α samples exceeded the range. Observations > ± 3.5 standard deviations (SD) away from the mean were winsorized to the nearest 0.01 pg/mL. No outliers were found in CRP or IL‐1β. For IL‐6, 0.06% (*n* = 2) of samples were below and 0.78% (*n* = 26) above 3.5 SD; for IL‐8, 0.03% (*n* = 1) were below and 0.09% (*n* = 3) above; and for TNF‐α, 0.09% (*n* = 3) were below and 0.15% (*n* = 5) above. All log‐transformed and winsorized biomarkers approached normality, with skewness values ranging from minimal (IL‐1*β* = 0.32, IL‐8 = 0.34, TNF‐α = 0.24, CRP = 0.49) to moderate (IL‐6 = 0.83) and kurtosis values close to 3 (IL‐1*β* = 2.39, IL‐6 = 4.47, IL‐8 = 2.80, TNF‐α = 3.32, CRP = 2.39).

Reliability of salivary biomarkers is reported in Supporting Information S1: Table S1. Reliability of salivary CRP across measurement days for study Years 1 and 2 was good (ICC = 0.80 and 0.84). The reliability of salivary cytokines ranged from fair to excellent (ICC 0.41–0.94). Prior to aggregating Year 2 and Year 3 data into a single follow‐up value, equality of variance was assessed. For all salivary biomarkers, Wilcoxon rank‐sum tests indicated no significant differences in measured levels at Year 2 and Year 3, while Levene's Tests confirmed equality of variance (Supporting Information S1: Table S2; *p* > 0.05 in all cases).

Bivariate Spearman's ⍴ correlations indicated that all five salivary biomarkers were positively associated with each other (*p* < 0.001 in all cases, Table 2). Salivary cytokines were more strongly associated with each other (*⍴* = 0.688–0.901) than with salivary CRP (*⍴* = 0.290–0.320). Salivary biomarker values stratified by BMI weight category and study year are reported in Supporting Information S1: Table S3.

**TABLE 2:** Means, standard deviations, and Spearman's ⍴ correlation coefficients with confidence intervals.

Variable	*M*	SD	1	2	3	4	5
Year 1							
1. BMIz	1.08	1.14					
2. CRP^a^	5.46	1.63	0.44**				
3. IL‐1β^a^	4.34	1.00	−0.12	0.29**			
4. IL‐6^a^	1.57	0.95	−0.11	0.30**	0.69**		
5. IL‐8^a^	6.15	1.03	−0.08	0.32**	0.83**	0.77**	
6. TNF‐α^a^	1.27	0.93	−0.07	0.31**	0.83**	0.75**	0.90**
Follow‐up (Year 2/3)							
1. BMIz	1.26	1.10					
2. CRP^a^	5.52	1.54	0.43**				
3. IL‐1β^a^	4.74	1.33	−0.13	0.18*			
4. IL‐6^a^	1.30	0.96	−0.10	0.21**	0.70**		
5. IL‐8^a^	6.39	1.28	−0.15*	0.17*	0.85**	0.79**	
6. TNF‐α^a^	1.17	1.13	−0.13	0.20**	0.85**	0.81**	0.90**

Abbreviations: BMIz, standardized body mass index; CRP, C‐reactive protein; IL, interleukin; *M*, mean; SD, standard deviation; TNF, tumor necrosis factor.

^a^ Units are log (pg/mL).

^*^ *p* < 0.05.

^**^ *p* < 0.01.

### 3.2 Main Analyses

The autoregressive cross‐lagged path model of bidirectional relationships between BMIz and salivary cytokines of inflammation at baseline (Year 1) and follow‐up (Year 2/3) was just identified and therefore had perfect fit. Model results are presented in Figure 2. A single autoregressive cross‐lagged model was used, adjusting for all salivary inflammatory biomarkers. BMIz demonstrated high stability over time (*β* = 0.88, *p* < 0.001). CRP, IL‐1β, and TNF‐α showed moderate stability over time (*β* values ranging from 0.48 to 0.59, *p* ≤ 0.001), whereas IL‐6 (*β* = −1.42 *p* = 0.11) and IL‐8 (*β* = −0.08, *p* = 0.64) were not stable over time. After accounting for these autoregressive paths, greater BMIz at baseline was prospectively associated with greater salivary CRP at follow‐up (β = 0.24, *p* < 0.001). Greater salivary CRP at baseline was prospectively associated with greater BMIz at follow‐up (β = 0.09, *p* = 0.008). There were no associations between salivary cytokines and BMIz (*p* > 0.100 in all cases). Multi‐group modeling revealed that the prospective relationships between BMIz and salivary biomarkers of inflammation did not differ between females and males (Δ *χ* ^2^ (36) = 32.87 *p* = 0.62).

## 4 Discussion

This study aimed to elucidate the prospective bidirectional relationships between BMIz and salivary biomarkers of inflammation over a one‐to 2‐year period in a racially, ethnically, and socioeconomically diverse sample of early adolescents. After controlling for stability of BMI and inflammation markers as well as sociodemographic covariates, greater BMIz in early adolescence was associated with increased salivary CRP at follow‐up 1–2 years later. Higher salivary CRP at baseline was prospectively associated with greater BMIz at follow‐up. This prospective bidirectional relationship between salivary CRP and BMIz was consistent across males and females and indicates the presence of a self‐reinforcing cycle where adiposity and inflammation amplify and reinforce one another [4, 7, 8, 9]. However, causality cannot be implied from the observational nature of this study. By contrast, no prospective associations were found between BMIz and salivary cytokines (IL‐6, IL‐1β, IL‐8, TNF‐α).

The results are consistent with the established link between chronic low‐grade inflammation and obesity. Although adiposity was not directly measured in this study, higher BMIz values typically reflect higher adiposity in US adolescents [40]. Adipose tissue, particularly in individuals with higher BMIz, secretes IL‐6, TNF‐α, and other pro‐inflammatory cytokines, which stimulate the hepatic production of CRP [4, 41, 42]. Elevated CRP levels, in turn, reflect a state of systemic inflammation that interferes with metabolic processes, worsening insulin resistance and promoting further adiposity [11, 43].

This study highlights the practicality of salivary biomarkers as a noninvasive approach to monitoring obesity‐related inflammation in pediatric populations [23]. Traditional methods of assessing inflammation, such as blood draws, can be invasive and distressing for children and adolescents, potentially limiting their feasibility in large‐scale studies and regular monitoring [16]. In contrast, saliva collection can be performed frequently without significant discomfort or risk, making it an ideal medium for longitudinal research and routine health assessments in young populations [19].

A critical issue is determining how well salivary biomarkers represent systemic inflammation. A review of 14 studies found a moderate correlation between salivary and serum CRP levels, with an *R* ^2^ value of 0.53 and a standard deviation of 0.23 [44]. For adults, salivary CRP accurately identified high versus low plasma CRP levels based on the American Heart Association's recommended cutoff point of 3 mg/L [32]. These results support the validity of salivary CRP as a measure of systemic inflammation. The prospective association between salivary CRP and subsequent BMIz found in this study is corroborated by a previous study [20], which reported that salivary CRP levels were positively associated with future BMIz over seven years in adolescents 10–17 years of age. Taken together, these studies indicate that salivary CRP is a promising marker for systemic inflammation in children and adolescents with overweight or obesity.

This study did not find significant associations between BMIz and salivary cytokines (IL‐6, IL‐1β, IL‐8, TNF‐α). One possible reason is that salivary cytokines are not a reliable measure of systemic inflammation. Previous research suggests that, unlike salivary CRP, salivary cytokines levels may not correlate well with levels measured in blood samples [20, 45, 46]. Salivary cytokines may be more reflective of local inflammation in the oral cavity than systemic inflammation, as suggested by elevated salivary IL‐1β, IL‐6, and IL‐8 levels in the presence of gingivitis in otherwise healthy children and adolescents [47, 48]. Therefore, the mixed results for serum‐saliva correlations for cytokines [29] and the non‐existent or inconsistent relationships between BMI and salivary cytokines in youth [20] may be due to the confounding effects of increased production of salivary cytokines with poor oral health.

The age and developmental stage of the study participants at saliva collection may matter. During early adolescence, significant hormonal changes and growth spurts occur, which can influence inflammatory processes differently than in adults [12, 13]. The relationship between BMIz and systemic cytokines may become more pronounced later in adolescence or adulthood as hormonal levels stabilize. Additionally, the transient nature of cytokine responses to acute and chronic stimuli [49, 50] can lead to variability, making it challenging to detect consistent associations with BMIz.

About 55% of children with obesity retain their obesity through adolescence, and about 80% of adolescents continue to have obesity as they enter adulthood [2]. The escalating persistence of obesity from adolescence into adulthood stresses the necessity for preventive and early intervention strategies to interrupt the cycle of obesity and inflammation [51]. Chronic low‐grade inflammation is now widely recognized as a major pathway leading to chronic conditions such as diabetes, cancer, and cardiovascular disease. This type of inflammation often develops silently over many years and can start in childhood. Adopting salivary CRP for large‐scale screenings in childhood and adolescence could be an innovative public health strategy to identify individuals with inflammatory trajectories indicative of a heightened risk for inflammation‐related diseases. In this manner, initial broad‐based screening with salivary CRP may be useful in triaging individuals who would benefit from early and more targeted diagnostic measures.

At present, there are no established guidelines or norms for the use of salivary C‐reactive protein (CRP) as an indicator of disease risk. A key priority is to standardize sampling and analytical methods, including optimizing collection techniques (passive vs. active drool), accounting for time‐of‐day variations, controlling salivary flow rate, and determining an appropriate dilution factor to align salivary and serum concentrations. Furthermore, there is a need for prospective studies to determine if salivary CRP can predict future inflammation‐related complications in children and adolescents with obesity before it can be used to monitor the effectiveness of interventions.

This study was limited by not collecting information on some potential confounders, including oral health, pubertal status, menstrual phase, and comorbidity diagnoses, such as type 2 diabetes, asthma, and gingivitis, or current medications, which are known to be associated with salivary biomarker levels [48, 52, 53]. In addition, salivary flow rate, which can affect the detection and quantification of some salivary biomarkers [50, 54], was not assessed in this study. It is also important to note that adiposity was not directly assessed in this study and that BMI does not always accurately predict long‐term changes in adiposity and cardiometabolic outcomes [55]. High attrition rates hindered the ability to perform multi‐timepoint longitudinal analyses. Despite these limitations, this study offers important evidence supporting the utility of salivary CRP as a predictive biomarker for obesity‐related inflammation.

In conclusion, this study found prospective bidirectional relationships between BMIz and salivary CRP levels in a healthy community sample of early adolescents, reflecting a feed‐forward cycle in which adiposity and inflammation mutually amplify each other. These results underscore the potential of salivary CRP as a noninvasive biomarker for monitoring obesity‐related inflammation and predicting future obesity‐related complications in children and adolescents. This highlights the importance of early intervention strategies to break the cycle of obesity and inflammation, thereby improving long‐term health outcomes.

## Author Contributions

S.M. and R.R.E. were responsible for project conception and overall cohort design. K.M.K., C.A.O., and S.M. designed this study, analyzed the data, performed the statistical analyses, and wrote the manuscript. D.A.G. contributed to developing the methodology and interpreting the results. All authors provided a critical review of the manuscript and approved the final manuscript submission.

## Conflicts of Interest

In the interest of full disclosure, D.A.G. is the Chief Scientific and Strategy Advisor at Salimetrics LLC and Salivabio LLC. These relationships are managed by the policies of the committees on conflict of interest at the Johns Hopkins University School of Medicine and the University of California at Irvine.

## Supporting information

Supporting Information S1

## Data Availability

The data that support the findings of this study are available from the corresponding author upon reasonable request.
