# Supplementary material for: Prospective Associations Between Salivary Biomarkers of Inflammation and Body Mass Index in Adolescents
Source: Obes Sci Pract. 2025 Jun 21;11(3):e70081. doi: 10.1002/osp4.70081 (PMC12181989; doi:10.1002/osp4.70081)
Supplement: Supplementary file 1 — Supporting Information S1 [file OSP4-11-e70081-s001.docx]

**Supplemental Information**

**Prospective Associations Between Salivary Biomarkers of Inflammation and Body Mass Index in Adolescents**

Keri M. Kemp^1^, Catheryn A. Orihuela^2^, Douglas A. Granger ^3-4^, Retta R. Evans^5^, Sylvie Mrug^6^

^1^ Division of Gerontology, Geriatrics and Palliative Care, Department of Medicine, Heersink School of Medicine, University of Alabama at Birmingham, Birmingham, AL, USA

^2^ Department of Family and Community Medicine, Heersink School of Medicine, University of Alabama at Birmingham, Birmingham, Alabama, USA

^3^ Institute for Interdisciplinary Salivary Bioscience Research, University of California, Irvine, CA, USA

^4^ Department of Pediatrics, Johns Hopkins University School of Medicine, Baltimore, MD, USA

^5^ Department of Human Studies, School of Education and Human Sciences, The University of Alabama at Birmingham, Birmingham, AL, USA

^6^ Department of Psychology, University of Alabama at Birmingham, Birmingham, AL, USA

**Corresponding Author**: Keri M Kemp (kerikemp@uab.edu).

**Table S1.** Within subject variance in salivary biomarkers across 4 sampling days within a weak

| Biomarker | CV [95% CI] | ICC [95% CI] |
| --- | --- | --- |
|  |  |  |
| **Year 1** |  |  |
| CRP (pg/mL) | 0.439 [0.403 - 0.474] | 0.799 [0.799 - 0.904] |
| IL-1β (pg/mL) | 0.461 [0.429 - 0.493] | 0.407 [0.360 - 0.834] |
| IL-6 (pg/mL) | 0.477 [0.444 - 0.509] | 0.469 [0.323 - 0.953] |
| IL-8 (pg/mL) | 0.526 [0.490 - 0.562] | 0.579 [0.599 - 0.803] |
| TNF-α (pg/mL) | 0.508 [0.475 - 0.541] | 0.409 [0.331 - 0.896] |
|  |  |  |
| **Year 2** |  |  |
| CRP (pg/mL) | 0.449 [0.390 - 0.508] | 0.839 [0.815 - 0.951] |
| IL-1β (pg/mL) | 0.509 [0.455 - 0.563] | 0.987 [0.608 - 0.999] |
| IL-6 (pg/mL) | 0.482 [0.426 - 0.538] | 0.938 [0.280 - 0.996] |
| IL-8 (pg/mL) | 0.583 [0.514 - 0.653] | 0.899 [0.630 - 0.963] |
| TNF-α (pg/mL) | 0.549 [0.486 - 0.612] | 0.885 [0.354 - 0.982] |
|  |  |  |

Abbreviations: CV, Coefficient of Variation; ICC, Intraclass Correlation Coefficient; CI, confidence interval; CRP, C-reactive protein; IL, interleukin; TNF, tumor necrosis factor

**Table S2.** Comparison of salivary biomarkers across Years 2 and 3

|  | Year 2 | | Year 3 | | Paired Wilcoxon  Signed-Rank Test | Levene's Test for  Homogeneity of Variance |
| --- | --- | --- | --- | --- | --- | --- |
| Biomarker | Median (Q1, Q3) | Min, Max | Median (Q1, Q3) | Min, Max |  |  |
|  |  |  |  |  |  |  |
| Log CRP (pg/mL) | 5.26 (4.21, 6.38) | 2.97, 8.99 | 5.65 (4.41, 6.85) | 2.97, 8.99 | *V* = 1435, *p* = 0.119 | *F*(1,261) = 0.069, *p*= 0.793 |
| Log IL-1β (pg/mL) | 4.57 (3.54, 5.60) | 0.91, 8.05 | 5.05 (3.77, 6.06) | 2.39, 8.05 | *V =* 1814*, p =* 0.954 | *F*(1,262) = 0.111, *p*= 0.740 |
| Log IL-6 (pg/mL) | 1.16 (0.67, 1.97) | -1.27, 5.10 | 1.16 (0.40, 1.76) | -1.90, 4.14 | *V* = 2194, *p* = 0.068 | *F*(1,260) = 0.102, *p*= 0.750 |
| Log IL-8 (pg/mL) | 6.12 (5.31, 7.31) | 2.39, 10.11 | 6.44 (5.54, 7.87) | 3.24, 10.01 | *V* = 1558, *p* = 0.239 | *F*(1,262) = 1.291, *p*= 0.257 |
| Log TNF-α (pg/mL) | 1.16 (0.29, 2.02) | -2.41, 4.84 | 1.03 (0.33, 1.95) | -1.24, 3.34 | *V* = 2254, *p* = 0.062 | *F*(1,262) = 0.724, *p*= 0.396 |
|  |  |  |  |  |  |  |

Abbreviations: Q, quartile; CRP, C-reactive protein; IL, interleukin; TNF, tumor necrosis factor

**Table S3.** BMI and salivary biomarker levels Median (Q1, Q3) stratified by BMI category and assessment year

|  | Healthy Weight | Overweight | Obesity | Severe Obesity | Very Severe Obesity |
| --- | --- | --- | --- | --- | --- |
|  |  |  |  |  |  |
| **Year 1** |  |  |  |  |  |
| BMI (kg/m^2^) | 18.6  (17.5, 20.1) | 22.6  (22.0, 23.4) | 26.3  (25.8, 27.2) | 31.9  (31.2, 32.7) | 38.3  (36.4, 41.4) |
| CRP (pg/mL) | 78.3  (38.2, 369.8) | 188.0  (62.8, 500.3) | 371.3  (159.5, 1,051.6) | 696.8  (187.6, 3,440.6) | 990.4  (522.1, 3,042.9) |
| IL-1β (pg/mL) | 80.6  (41.8, 161.9) | 62.8  (35.5, 130.2) | 96.0  (41.4, 171.1) | 53.6  (29.9, 108.9) | 56.5  (29.7, 93.2) |
| IL-6 (pg/mL) | 4.7  (2.6, 9.0) | 3.8  (2.2, 7.0) | 4.1  (2.8, 8.7) | 3.4 (2.5, 7.5) | 3.0  (2.1, 5.4) |
| IL-8 (pg/mL) | 411.4  (223.6, 991.5) | 330.7  (185.8, 793.9) | 542.0  (281.9, 1,147.5) | 407.5  (249.6, 521.4) | 272.5  (185.3, 364.5) |
| TNF-α (pg/mL) | 3.3  (1.9, 6.6) | 2.8  (1.8, 6.2) | 4.0  (2.6, 7.1) | 3.3  (1.8, 5.4) | 2.2  (1.6, 2.7) |
|  |  |  |  |  |  |
| **Year 2** |  |  |  |  |  |
| BMI (kg/m^2^) | 20.5  (19.1, 21.5) | 23.5  (23.2, 25.3) | 28.0  (26.8, 28.5) | 32.8  (32.1, 34.8) | 39.9  (36.4, 41.3) |
| CRP (pg/mL) | 73.9  (39.0, 336.3) | 170.3  (47.1, 336.8) | 280.6  (96.7, 620.5) | 358.0  (236.2, 2,077.2) | 1,083.0  (452.2, 3,934.3) |
| IL-1β (pg/mL) | 102.1  (34.5, 251.2) | 93.1  (45.9, 180.8) | 144.1  (30.2, 242.1) | 76.9  (41.4, 148.2) | 70.9  (39.2, 203.3) |
| IL-6 (pg/mL) | 3.1  (1.9, 8.8) | 3.5  (2.0, 5.8) | 2.8  (1.9, 7.3) | 2.5  (1.6, 4.0) | 3.2  (2.3, 5.3) |
| IL-8 (pg/mL) | 485.1  (224.6, 1,968.0) | 464.4  (248.4, 1,271.9) | 469.7  (167.2, 1,261.7) | 477.8  (200.2, 561.4) | 364.3  (164.1, 985.4) |
| TNF-α (pg/mL) | 3.2  (1.4, 7.8) | 3.7  (1.6, 8.0) | 3.8  (1.2, 8.3) | 2.3  (1.5, 3.7) | 1.9  (1.2, 4.3) |
|  |  |  |  |  |  |
| **Year 3** |  |  |  |  |  |
| BMI (kg/m^2^) | 20.2  (19.0, 21.4) | 24.9  (24.3, 25.3) | 29.0  (27.9, 30.1) | 33.3  (32.1, 34.0) | 40.4  (39.5, 43.1) |
| CRP (pg/mL) | 222.9  (71.2, 758.2) | 168.7  (69.4, 236.5) | 457.1  (192.6, 789.7) | 700.6  (578.9, 1,613.7) | 1,679.4  (493.2, 3,738.9) |
| IL-1β (pg/mL) | 168.6  (42.4, 494.4) | 97.7  (19.3, 430.3) | 161.9  (132.5, 356.5) | 174.4  (40.8, 345.6) | 92.9  (55.7, 229.6) |
| IL-6 (pg/mL) | 3.5  (1.4, 6.9) | 3.7  (1.6, 6.7) | 3.0  (2.4, 4.7) | 3.2  (1.4, 4.5) | 2.2  (1.4, 6.0) |
| IL-8 (pg/mL) | 664.6  (246.4, 2,971.2) | 416.3  (247.8, 2,591.2) | 902.9  (325.8, 1,366.8) | 350.6  (186.5, 3,215.4) | 563.4  (192.8, 978.3) |
| TNF-α (pg/mL) | 2.8  (1.3, 9.5) | 2.5  (1.2, 7.2) | 3.4  (2.5, 4.4) | 2.6  (0.8, 5.0) | 2.7  (1.6, 5.3) |

Abbreviations: Q, quartile; BMI, body mass index; CRP, C-reactive protein; IL, interleukin; TNF, tumor necrosis factor
